# Pain Reduction With an Immersive Digital Therapeutic in Women Living With Endometriosis-Related Pelvic Pain: At-Home Self-Administered Randomized Controlled Trial

**DOI:** 10.2196/47869

**Published:** 2023-06-28

**Authors:** Benjamin Merlot, Valéry Elie, Adrien Périgord, Zoé Husson, Amandine Jubert, Isabella Chanavaz-Lacheray, Thomas Dennis, Maryne Cotty-Eslous, Horace Roman

**Affiliations:** 1 Franco European Multidisciplinary Endometriosis Institute Clinique Tivoli-Ducos Bordeaux France; 2 Lucine Bordeaux France

**Keywords:** randomized controlled trial, virtual reality, digital therapeutics, digital health, endometriosis, chronic pain, pelvic pain, women's health, digital health intervention, patient outcome

## Abstract

**Background:**

The management of chronic pelvic pain in women with endometriosis is complex and includes the long-term use of opioids. Patients not fully responsive to drugs or ineligible for surgical treatments need efficient alternatives to improve their quality of life and avoid long-term sequelae.

**Objective:**

This randomized controlled trial aimed to assess the effects of repeated at-home administrations of a 20-minute virtual reality (VR) solution (Endocare) compared with a sham condition on pain in women experiencing pelvic pain due to endometriosis.

**Methods:**

Patients were instructed to use the VR headsets twice daily for at least 2 days and for up to 5 days starting on their first day of painful periods. Pain perception was measured using a numerical scale (0-10) before and 60, 120, and 180 minutes after each treatment administration. General pain, stress, fatigue, medication intake, and quality of life were reported daily by patients.

**Results:**

A total of 102 patients with endometriosis were included in the final analysis (Endocare group: n=51, 50%; sham group: n=51, 50%). The mean age was 32.88 years (SD 6.96) and the mean pain intensity before treatment was 6.53 (SD 1.74) and 6.22 (SD 1.69) for the Endocare group and the sham control group, respectively (*P*=.48). Pain intensity decreased in both groups from day 1 to day 5 along with a decrease in medication use. Maximum pain intensity reduction of 51.58% (SD 35.33) occurred at day 2, 120 minutes after treatment for the Endocare group and of 27.37% (SD 27.23) at day 3, 180 minutes after treatment for the control group. Endocare was significantly superior to the sham on day 1 (120 minutes, *P*=.04; 180 minutes, *P*=.001), day 2 (0 minutes, *P*=.02; 60, 120, and 180 minutes, all *P*<.001), and day 3 (60 minutes, *P*=.01; 120 minutes, *P*=.005; 180 minutes, *P*=.001). Similarly, the mean perceived pain relief was significantly higher with Endocare on day 1 (120 and 180 minutes *P*=.004 and *P*=.001, respectively) and day 2 (60, 120, and 180 minutes *P*=.003, *P*=.004, and *P*=.007, respectively) compared to the control. No adverse event was reported.

**Conclusions:**

This study confirmed the effectiveness and safety of self-repeated administrations of a VR immersive treatment used at home while reducing overall pain medication intake in women diagnosed with endometriosis experiencing moderate-to-severe pelvic pain.

**Trial Registration:**

ClinicalTrials.gov NCT05172492; https://clinicaltrials.gov/ct2/show/NCT05172492

## Introduction

Chronic pelvic pain is the main symptom of women suffering from endometriosis [1]. Severely affecting quality of life, this pain can be constant and occur in attacks triggered by various conditions such as menstruation (dysmenorrhea). Endometriosis-related pain negatively impacts sexual, family, and work life [2].

Endometriosis is characterized by the occurrence of lesions with behavior comparable to that of the endometrium, including cyclic bleeding during periods, followed by inflammation and proliferation of the conjunctive and muscular cells outside the uterus, mainly on the pelvic peritoneum, ovaries, and rectovaginal septum [3]. Endometriosis is a multifactorial disease resulting from the combined action of genetic and environmental factors [4,5] and is a major contributor to infertility [6]. The prevalence of endometriosis in women with chronic pelvic pain ranges from 2% to 74% and is over 33% in women with acute pelvic pain [7].

The most common painful symptom of endometriosis is dysmenorrhea (80%), followed by deep dyspareunia (30%) [8]. These pains can be nociceptive, neuropathic, or a combination of both [9]. Thus, the relationship between pain and the characteristics of endometriosis is complex [10], and current management of pelvic pain involves medical and/or surgical treatments [11,12].

Medical treatments, including combined hormonal contraceptives, progestin, and gonadotropin-releasing hormone agonists, are generally effective in treating endometriosis-related pain [13-15], while inhibiting ovulation and suppressing menstruation [8]. However, they do not lead to the disappearance of endometriosis lesions, their efficiency is reduced in the case of deep lesions, and they have a contraceptive effect, which renders them not suitable in women with pregnancy intention in which only limited analgesic treatments can be employed [8].

Surgery is usually not considered as a first-line treatment [8] but is rather only indicated in cases of the failure of pain relief using medical treatment, as well in various cases of infertility. However, recurrence of pain after surgery, particularly in women without additional postoperative hormonal therapy, is frequent; recurrence rates of postoperative endometriosis after 2 to 5 years vary depending on the localization of the disease, ranging between 30% and 50% [16,17].

Therefore, the management of patients with endometriosis who are not eligible, are refractory, or not fully responsive to hormonal therapies or drug treatments, or are ineligible for surgical treatment (ie, awaiting pregnancy or not recommended for surgery) remains a problem. Accordingly, nonpharmacological alternatives are urgently needed to increase the chronic pelvic pain therapeutic arsenal.

Studies have shown that virtual reality (VR) is an effective mode to reduce acute pain such as that experienced by burn victims, in cases of lumbar puncture, women during labor, or dental surgery [18-21], as well as for chronic pain such as musculoskeletal pain, neuropathic pain, and cancer [22,23]. However, most studies on VR for pain have been conducted in a medical context under the supervision of a health care team [24-27] and only few are conducted at patients’ homes with treatment self-administered by the patients themselves [28]. In fact, most of the medical devices developed to treat pain, either acute or chronic, are designed to be used by medical staff in a medical setting [24,27,29-32]**.**

Endocare is a class I medical device, or digital therapeutic (DTx), consisting of visual and auditory therapeutic procedures administered using a VR headset to treat pelvic-perineal pain in patients with endometriosis at home. Promising results of the use of Endocare were reported in a single-dose pilot study conducted in a controlled hospital setting in women with pelvic-perineal pain of ≥4/10 intensity: the study treatment provided pain relief and decreased pain intensity by a maximum of 42% compared with only 22% in the control group (*P*=.04), and was effective for up to 4 hours after administration [24].

Considering that chronic pelvic pain should be treated at home and not only in a medical environment, this study was conducted to evaluate the efficacy and safety of a VR treatment (Endocare) to reduce chronic pelvic pain when used at home in full autonomy without medical supervision compared to a digital control in a double-blinded, randomized, sham-controlled clinical trial. The objective of the study was to evaluate the effects of Endocare compared to a digital control on the mean change in pain intensity 1, 2, and 3 hours after daily use during the 5 most painful consecutive days of the month in women with chronic pelvic-perineal pain associated with endometriosis.

## Methods

### Study Design

The “Endocare for Pelvic-perineal Pain Related to Endometriosis Used at Home” study was a randomized, sham-controlled, double-blinded, two-parallel-group, single-site, interventional clinical trial evaluating the efficacy of software as a medical device (SaMD) product on reducing endometriosis-related pain at home compared to a digital control. The study was conducted between December 2021 and October 2022 at the Franco-European Multidisciplinary Endometriosis Institute, Tivoli-Ducos Clinic in Bordeaux, France.

### Study Population

Patients included in the study were women over 18 years old with a diagnosis of endometriosis and/or adenomyosis; willing to participate in the study; and women with at least 2 consecutive days per month of endometriosis-related pelvic-perineal pain of moderate to severe intensity (numerical scale≥4), including women without amenorrhea (pain around the onset of menstruation) and women with amenorrhea (in which the most intense pain of the month should last for at least 2 days). Pregnant or nursing women were not eligible for inclusion in the study, along with women who were participating or had participated in an interventional study within the last 30 days before inclusion. Those with severe visual, auditory, or cognitive impairment; color blindness, photosensitivity, epilepsy, or motion sickness; women whose pain is occasional and not present at each menstrual period; those who had previously received VR treatments; and women under judicial protection, guardianship, curatorship, or a protective mandate were also excluded.

### Study Outcomes

The impact of the study treatment on patients’ pain relief and quality of life was evaluated using a visual analog scale (stress) [33], Pichot scale (fatigue) [34], the Endometriosis Health Profile (EHP-5) endometriosis-specific quality of life questionnaire [35], and the Pain Catastrophizing Scale (PCS) [36].

Treatment adherence, tolerance, Patient's Global Impression of Change (PGIC) [37], and patients’ satisfaction were also assessed.

### Study Devices

Endocare is a class I software medical device developed by Lucine (Bordeaux, France) associated with a VR headset intended to decrease pelvic pain related to endometriosis. The software consists of a 20-minute treatment combining auditory and visual therapeutic stimulations integrated in a 3D VR environment, including binaural beats, verbal hypnotic injunction, nature-based sounds, distraction of attention, and bilateral alternative stimulations.

The digital control (control) was also developed by Lucine as a 20-minute audio-video composition similar to Endocare (same context, environment, and duration) with exposure to nature sounds, but without Endocare’s stimulations, as previously described [24]. The sham control and Endocare groups used the same hardware: Oculus Quest 2 VR headsets and AKG K-240 MKII audio headphones.

### Study Procedures

Upon inclusion, eligible patients were randomized to either the Endocare or control arm, were given the study devices and a follow-up diary, and instructed on how to use these materials during their 5 days of participation. Patients were asked to use the treatment starting on their first day of painful periods (next cycle after study inclusion) and up to twice a day with a minimum of 3 hours between exposures. Patients were given the possibility to stop their study treatment after 2 days in case of pain relief and to continue using their usual medications while reporting their uses in their follow-up diary (drug name, dose, frequency).

For the baseline assessment, once enrolled, patients were invited to complete 2 questionnaires to assess their baseline profiles: EHP-5 questionnaire and PCS. The 11 items from the EHP-5 questionnaire were rated from “never” to “always,” while dramatization (PCS) was evaluated on a 5-point Likert-type scale ranging from 0 (not at all) to 4 (all the time).

Subsequently, on their first day of pain, patients informed the investigating staff of their treatment initiation (day 1) and started completing their daily follow-up diary for 5 days.

### Measures

#### Pain Intensity and Pain Relief

Pain intensity was evaluated on an 11-point numeric rating scale (0=no pain, 10=unbearable pain) at wakeup, before and after each treatment (60, 120, and 180 minutes), and at bedtime. Pain relief was evaluated on a 5-point categorical scale (0=no relief, 1=slight relief, 2=moderate relief, 3=important relief, and 4=complete relief) after each treatment at 60, 120, and 180 minutes. Patients were also asked to report any adverse event that could have occurred during their 5 days of participation.

#### Concomitant Medications

Participants were allowed the use of pain medications in case of an insufficient analgesic effect of the study treatment. In such cases, they were invited to report their medication intake (drug name, dose, frequency) in their follow-up diaries.

#### Fatigue and Stress

Fatigue and stress were reported by patients twice a day at wakeup and bedtime, using the Pichot scale with 8 items ranging from 0 to 4 (0=not at all, 1=a little, 2=moderately, 3=a lot, 4=extremely) [34] and the visual analog scale consisting of a small unmarked 100-mm ruler with the ends labeled “none” and “as bad as possible,” respectively [33].

#### End of Study

On day 6, patients were contacted by the study staff to perform a remote end-of-study visit by telephone. Patients were invited to complete different questionnaires: the EHP-5 after study participation, the PGIC, and to rate their global level of satisfaction.

### Statistics

The sample size was calculated based on data from the “Single Care” pilot study [24]. Considering a mean difference of 0.9 between groups to be observed, SD of 1.54, and a dropout rate of 20%, 120 patients had to be recruited (60 patients per treatment arm, randomization 1:1, power=0.80, α=.05). All statistical analyses were performed using R software.

Descriptive statistics were used to describe the sample with respect to demographics, medical history, and pain history. Parametric Student *t*-tests and ANOVA were used to compare means between groups, and the Pearson test was used to evaluate the correlation between PCS score and pain intensity at 60 minutes after treatment on day 1. Differences between the percentages of patients achieving defined targets of pain reduction were analyzed using bilateral proportion tests. For categorical variables, independence between treatment arms was evaluated using χ^2^ and Fisher-exact tests, depending on the sample sizes. Differences in pain intensity between the two groups were assessed using a linear mixed model for repeated measures with a 2×2 design (nlme R package), including the effects of group (Endocare vs control), time (before, and 60, 120, and 180 minutes after treatment for each day of the treatment), along with a random effect on the intercept of each participant. Evaluation of the group×time interaction served as a measure of the efficacy of the study treatment. A contrast analysis (emmeans package) was also performed to test the differences before and after treatments (at all time points) in both groups. Differences in pain intensity, fatigue, and stress were also assessed using a linear mixed model for repeated measures (2×2 design) at wakeup and bedtime on days 1 and 2. *P* values were adjusted using the Tukey method. Missing data at several time points were not replaced. False discovery rate corrections were used to adjust for multiple testing when needed.

### Ethical Considerations

The study was conducted in compliance with the 1964 Declaration of Helsinki and later amendments, ISO 14155:2020, Medical Device Regulation (EU) 2017/745, and ICH-GCP E6. The study received approval on October 26, 2021, by the Comité de Protection des Personnes, Région Ouest, Ouest 1 (approval number 2021-A02358-33). All patients signed an informed consent form before inclusion and performing any study-related procedure. The study is registered at ClinicalTrials.gov under registration number NCT05172492.

## Results

### Baseline Study Population

Of the 120 patients screened, 120 were randomized to the Endocare (n=60) and control (n=60) arms, and 103 completed the study. As study procedures were set to start within 1 month following patients’ inclusion, 14 patients prematurely ended their participation after randomization: 11 experienced no more pain before the start of the study (noninclusion criteria) and 3 became pregnant (exclusion criteria). Additionally, 1 patient did not perform the study procedures due to a lack of time. Two patients were considered as lost to follow-up and 1 patient did not return her questionnaire after completing her participation.

Thus, 102 of the 120 recruited patients (85.0%) were included in the study analyses, including 51 in the Endocare group and 51 in the control group. Patients’ demographic characteristics were similar between groups, with the exception of weight (Table 1). Albeit slightly statistically significant (*P*=.03), the weight difference between groups was deemed inconsequential, as this parameter was not correlated to any aspect of the treatment.

The patients’ medical history was also similar between the groups, enabling effective comparison (Tables 2 and 3). Considering the overall cohort of 102 participants, the majority experienced chronic pelvic pain, followed by dysmenorrhea, dyspareunia, dyschezia, and dysuria. Moreover, over one-third (n=36/102) of patients had adenomyosis (Table 2).

Due to their endometriosis and the pain related to their condition, most of the patients declared using chronic drug treatments at baseline, including analgesics, antiemetics, and antinauseants; anti-inflammatory and antirheumatic products; and drugs for gastrointestinal disorders (Table 3). No difference was observed in the frequency of use of these drugs between the two study groups.

**Table 1:** Study population demographic characteristics at baseline.

Characteristics		Total (N=102)	Control (n=51)	Endocare (n=51)	*P* value	
**Age (years)**						
	Mean (SD)	32.9 (6.96)	32.1 (7.3)	33.7 (6.6)	.26	
	Median (range)	33 (18-49)	33 (18-47)	34 (19-49)	N/A^a^	
**Height (cm)**						
	Mean (SD)	163.9 (6.56)	163.9 (6.82)	163.8 (6.35)	.89	
	Median (range)	163.5 (149-182)	163 (149-182)	164 (150-178)	N/A	
**Weight (kg)**						
	Mean (SD)	64.6 (12.26)	61.9 (10.95)	67.3 (13.00)	.03	
	Median (range)	62.5 (42-96)	59 (42-92)	66 (43-96)	N/A	

^a^N/A: not applicable.

**Table 2:** History of endometriosis.

Characteristics			Total (N=102)		Control (n=51)		Endocare (n=51)		*P* value	
**Time since endometriosis diagnosis (months)**										
	Mean (SD)	25.6 (39.21)		23.1 (38.48)		28.2 (40.15)		.51		
	Median (range)	6 (0-184)		5 (0-149)		8 (0-184)		N/A^a^		
**Type of endometriosis, n (%)**										
	Superficial	14 (13.7)		9 (17.6)		5 (9.8)		.39		
	Peritoneal	13 (12.7)		9 (17.6)		4 (7.8)		.23		
	Cystic or ovarian endometriosis	40 (39.2)		20 (39.2)		20 (39.2)		>.99		
	Deep infiltrating endometriosis	84 (82.4)		38 (74.5)		46 (90.2)		.07		
	Digestive locations	0 (0.0)		0 (0.0)		0 (0.0)		>.99		
	Other	0 (0.0)		0 (0.0)		0 (0.0)		>.99		
Presence of adenomyosis, n (%)			36 (35.3)		16 (31.4)		20 (39.2)		.54	
History of surgical management for endometriosis, n (%)			22 (21.6)		8 (15.7)		14 (27.5)		.23	
**Time since the last surgery (months)**										
	Missing, n (%)	20 (82)		8 (43)		12 (39)		N/A		
	Mean (SD)	38.5 (46.36)		41.5 (56.23)		36.5 (41.09)		.83		
	Median (range)	16.5 (1-146)		6 (1-146)		24 (1-137)		N/A		
**Current management of endometriosis, n (%)**										.83
	Hormonal treatment	41 (40.2)		19 (37.3)		22 (43.1)				
	Hormonal intrauterine device	4 (3.9)		2 (3.9)		2 (3.9)				
	None	57 (55.9)		30 (58.8)		27 (52.9)				
**Usual pain symptoms**										
	Chronic pelvic pain	96 (94.1)		46 (90.2)		50 (98.0)		.21		
	Dysmenorrhea	78 (76.5)		42 (82.4)		36 (70.6)		.24		
	Dysuria	17 (16.7)		7 (13.7)		10 (19.6)		.60		
	Dyschezia	26 (25.5)		9 (17.6)		17 (33.3)		.11		
	Dyspareunia	74 (72.5)		40 (78.4)		34 (66.7)		.27		

^a^N/A: not applicable.

**Table 3:** Medication use at baseline.

Medications		Total (N=102), n (%)	Control (n=51), n (%)	Endocare (n=51), n (%)	*P* value
**Analgesics**					
	Anilides	15 (14.7)	10 (19.6)	5 (9.8)	.26
	Natural opium alkaloids	3 (2.9)	1 (2.0)	2 (3.9)	>.99
	Opioids in combination with antispasmodics	2 (2.0)	0 (0.0)	2 (3.9)	.50
	Opioids in combination with nonopioid analgesics	8 (7.8)	5 (9.8)	3 (5.9)	.72
	Other analgesics and antipyretics	10 (9.8)	5 (9.8)	5 (9.8)	>.99
	Other opioids	3 (2.9)	0 (0.0)	3 (5.9)	.24
	Salicylic acid and derivatives	1 (1.0)	1 (2.0)	0 (0.0)	>.99
Other antiemetics and antinauseants		1 (1.0)	0 (0.0)	1 (2.0)	>.99
Other antiepileptics		0 (0.0)	0 (0.0)	0 (0.0)	>.99
**Anti-inflammatory and antirheumatic products**					
	Fenamates	0 (0.0)	0 (0.0)	0 (0.0)	>.99
	Propionic acid derivatives	26 (25.5)	12 (23.5)	14 (27.5)	.82
Proton pump inhibitors (for acid-related disorders)		0 (0.0)	0 (0.0)	0 (0.0)	>.99
Other drugs for functional gastrointestinal disorders		6 (5.9)	4 (7.8)	2 (3.9)	.68

### Adherence to Study Protocol

On study day 1, 102 patients used both study treatments and correctly filled in their follow-up diaries. Study device use started to decrease on day 2 (Endocare: n=49/51, 96%; control: n=49/51, 96%), significantly decreased on day 3 (Endocare: n=34/51, 67%; control: n=30/51, 59%), and decreased even more on day 4 (Endocare: 24/51, 47%; control: n=21/51, 41%) and day 5 (Endocare: n=18/51, 35%; control: n=18/51, 35%). Some participants who stopped using the study treatments continued to complete their follow-up diary until the end of the study period.

### Pain Assessment

#### Overview

Patients were advised to use the treatment (ie, Endocare or control) starting on the first painful day of their cycle. The use of the study treatment was mandatory for at least 2 days (ie, days 1 and 2), and patients had the possibility of continuing the treatment for the next 3 days if they experienced pain (ie, days 3, 4, and 5).

#### Pain Intensity at Days 1-2

**Figure 1:**
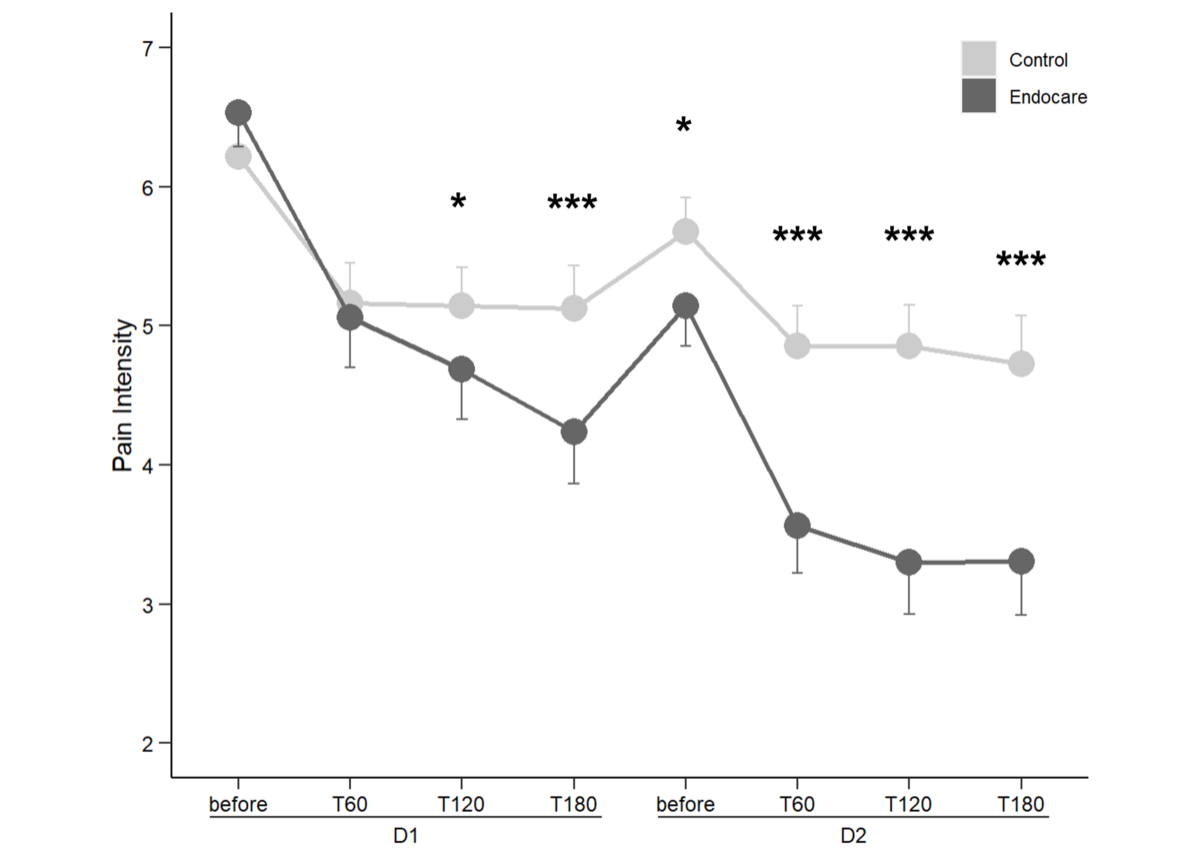
Comparison of pain intensity between treatment arms on the first 2 days of participation. D: day; T: time (minutes after treatment use). ***P<.001, **P<.01, *P<.05.

Differences in pain intensity between baseline (prior to treatment initiation on day 1) and after treatment (60, 120, and 180 minutes) over the first 2 days was assessed in the two groups and the results are summarized in Figure 1.

As the number of active treatment users was drastically reduced from day 3 onward, only the first 2 days were included in the mixed model. Moreover, no difference was observed in the number of daily self-administrations between the two groups. On days 1 and 2, the mean pain intensity decrease at 60 minutes was 1.48 (SD 1.68) in patients using Endocare and was 0.92 (SD 1.28) in the control group.

Compared to the initial (pretreatment) pain intensity levels, pain intensity reduction reached 51.6% (SD 35.34) at 120 minutes and 51.2% (SD 37.33) at 180 minutes on day 2 in the Endocare group versus 21.2% (SD 30.12) and 23.9% (SD 36.06), respectively, in the control group. Maximum pain intensity reduction was achieved on day 2 at 120 minutes after Endocare treatment administration.

No significant group effect was observed (*F*_100_=0.514, *P*=.48), but significant effects of time (*F*_668_=7.181, *P*<.001) and of the group×time interaction (*F*_668_=5.559, *P*<.001) were identified. Detailed analysis of interactions revealed significant group×time interactions at day 1, 120 minutes (*t*_668_=–2.090, *P*=.04); day 1, 180 minutes (*t*_668_=–3.297, *P*=.001); day 2, before treatment (*t*_668_=–2.303, *P*=.02); day 2, 60 minutes (*t*_668_=–4.147, *P*<.001); day 2, 120 minutes (*t*_668_=–4.719, *P*<.001); and day 2, 180 minutes (*t*_668_=–4.391, *P*<.001). In all cases, pain reduction from the baseline was significantly higher in the Endocare group from day 1 at 120 minutes to day 2 at 180 minutes after treatment compared to that of the control group (Figure 1). No difference was observed at day 1, 60 minutes (*t*_668_=–1.202, *P*=.23) between the two groups.

Contrast analysis revealed that pain intensity reduction was significant for each time point for the Endocare group compared to levels reported at day 1 before treatment and day 2 before treatment (*P*<.001). For the control group, except for the comparison between day 1 and day 2 before treatment (*t*_668_=2.019, *P*=.47), all other reductions were significant (day 1 60-180 minutes vs day 1 before treatment: *P*=.002, *P*=.001, and *P*=.001, respectively; day 2 60-180 minutes vs day 1 before treatment: all *P*<.001; day 2 60-180 minutes vs day 2 before treatment: *P*=.04, *P*=.04, and *P*=.008, respectively).

To illustrate the efficacy of the study treatments under test, the percentage of patients achieving 20%, 30%, 50%, and 100% pain reduction at day 2, 180 minutes after treatment compared to the baseline was calculated (Table 4). For each target of pain reduction, the percentage of patients was significantly higher in the Endocare group than in the control group (Table 4).

**Table 4:** Patients achieving defined targets of pain intensity reduction at day 2, 180 minutes after treatment.

Group	Pain intensity reduction from day 1 before treatment to day 2 180 min after treatment, n (%)			
	20%	30%	50%	100%
Control (n=47)	22 (46.8)	15 (31.9)	12 (25.5)	3 (6.4)
Endocare (n=46)	38 (82.6)	31 (67.4)	22 (47.8)	13 (28.3)
*P* value	<.001	<.001	.03	.005

#### Pain Intensity at Days 3-5

For the next 3 days, the number of participating patients significantly decreased when compared to that at day 1 (see “Adherence to Study Protocol” section above). Nevertheless, patients in the Endocare group reported significantly lower pain intensity at day 3 60, 120, and 180 minutes after treatment compared to the control group. No difference was observed on days 4 and 5.

**Figure 2:**
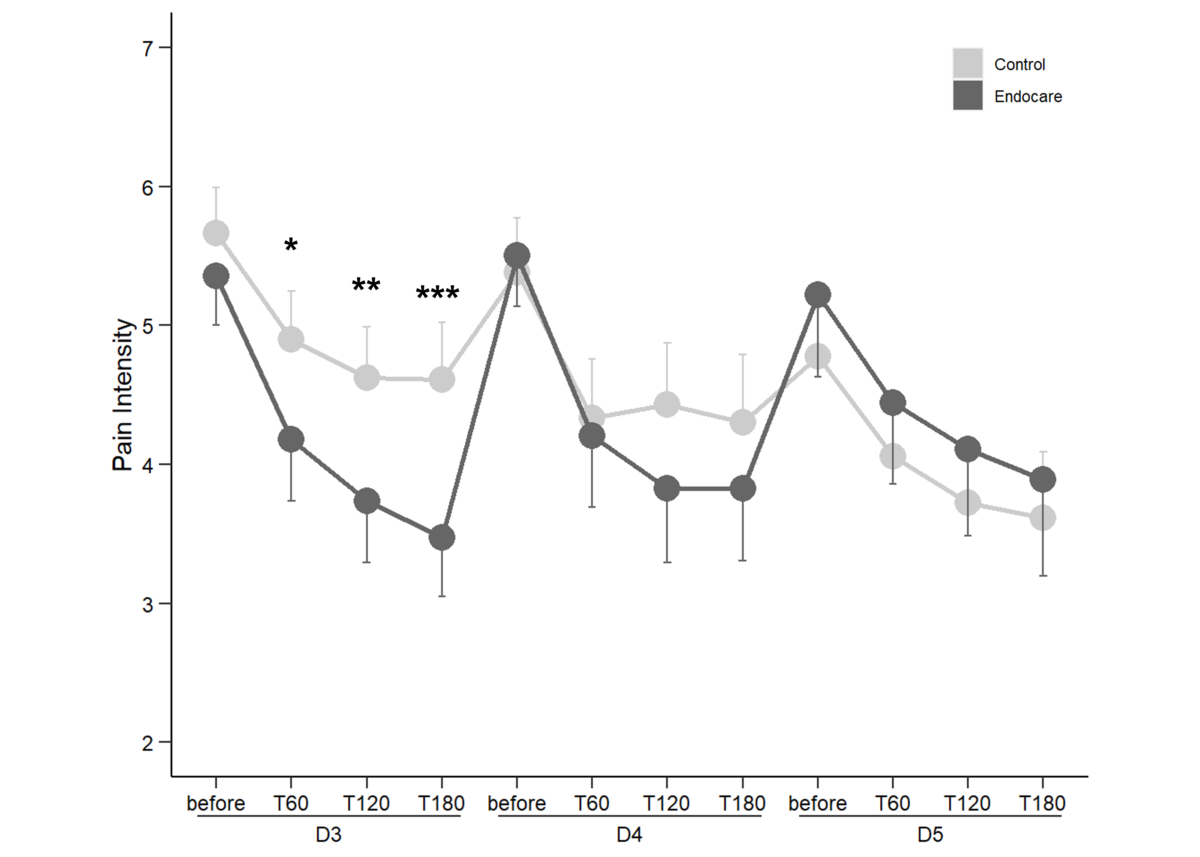
Comparison of pain intensity between treatment arms on days 3 to 5 of participation. D: day; T: time (minutes). ***P<.001, **P<.01, *P<.05.

Despite the decrease in the number of participants on day 3, compared to the initial (pretreatment) pain intensity levels, pain intensity reduction at 60, 120, and 180 minutes after treatment was significantly higher in the Endocare group at 24.2% (SD 33.59), 41.8% (SD 40.68), and 46.6% (SD 36.02), respectively, compared to that of the control group at 12.0% (SD 22.47), 23.4% (SD 29.43), and 27.4% (SD 27.23), respectively. No difference was observed between the groups on days 4 and 5 (Figure 2).

#### Daily Analysis

Patients were free to use the study treatment at any time of the day, but not more than twice daily. Therefore, measurements were also analyzed for each day independently to focus solely on the pain intensity reduction from the baseline to 180 minutes, without the influence of the varying delay between consecutive treatment uses.

**Figure 3:**
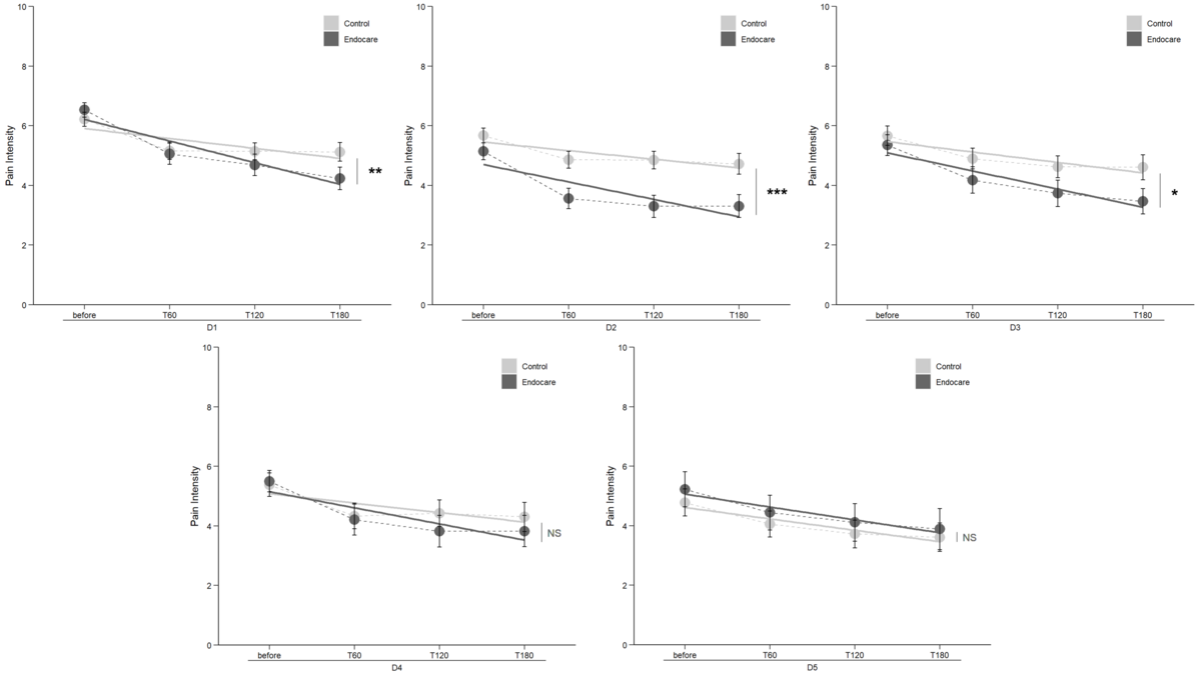
Comparison of pain intensity between treatment arms for each independent day (D1-D5). Mean pain intensity is represented by a dotted line and the linear regression is represented by a solid line. NS: not significant (*P*>.05); T: time after treatment (minutes). ***P<.001, **P<.01, *P<0.05.

From days 1 to 5, the mean pain intensity decrease at 60 minutes was 1.33 (SD 1.60) in the Endocare group compared to a 0.88 (SD 1.27) reduction in the control, and 30% of assessments reported a pain reduction of ≥40% 1 hour after the session compared to the daily baseline. Results of the comparison between both treatment arms for days 1-5 using a linear model for repeated measures are detailed in Figure 3. A significant pain intensity reduction was observed for day 1 (*P*<.001), day 2 (*P*<.001), and day 3 (*P*=.04). No significant difference was observed for days 4 and 5.

#### Wakeup and Bedtime

**Figure 4:**
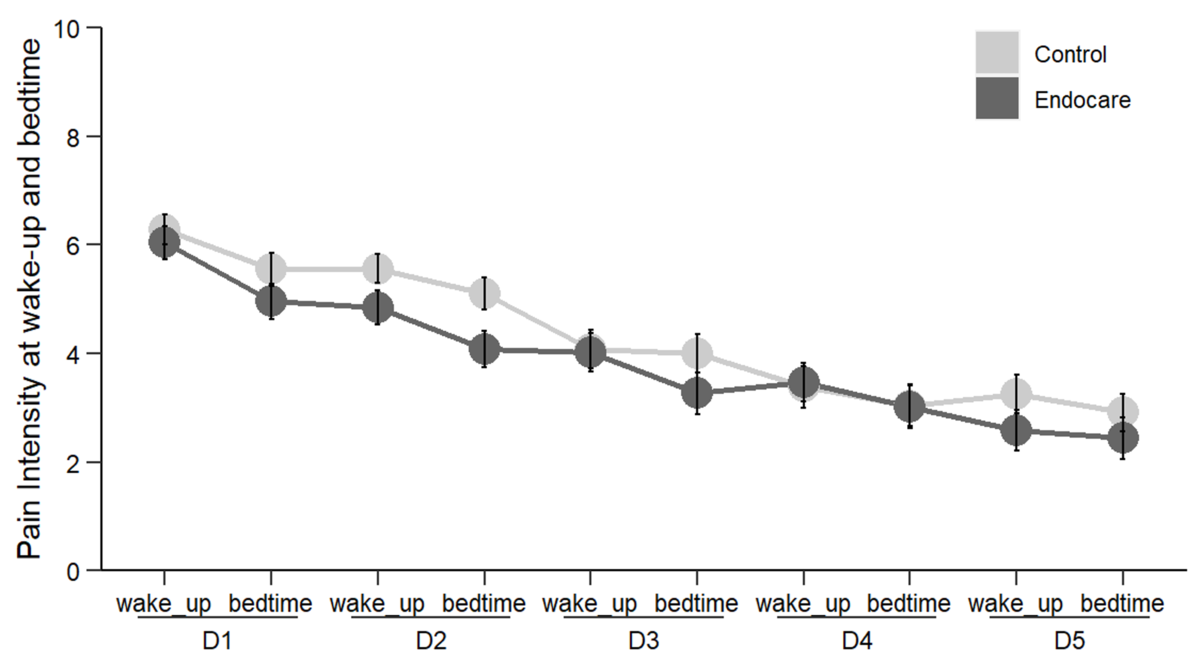
Pain intensity at wakeup and bedtime. D: day.

Pain intensity was also measured each day at wakeup and bedtime for the 5 days of the study and compared using mixed models (Figure 4). Our results did not demonstrate a significant effect of the group, neither for wakeup (*F*_100_=0.261, *P*=.61) nor for bedtime (*F*_99_=1.654, *P*=.20). In addition, there was no effect of the group×day interaction for wakeup (*F*_384_=0.957, *P*=.43) or bedtime (*F*_379_=0.755, *P*=.56). However, a significant effect of day was found for both wakeup (*F*_384_=28.720, *P*<.001) and bedtime (*F*_379_=20.769, *P*<.001). These results indicated a reduction in pain intensity at both wakeup and bedtime, but that this reduction was not different between the two groups.

#### Pain Relief

**Figure 5:**
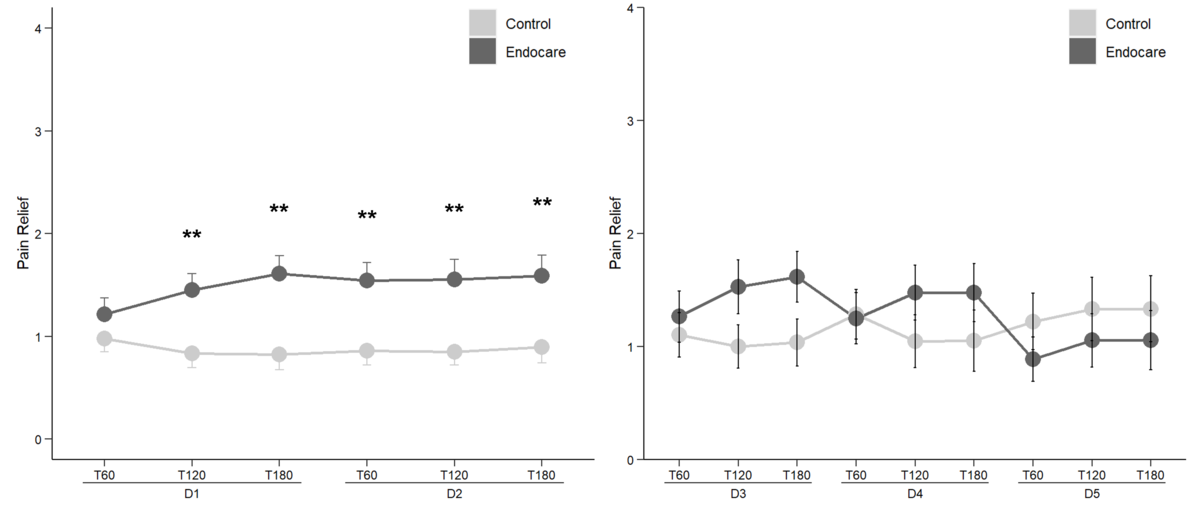
Comparison of pain relief between treatment arms from day 1 to 5 of participation. D: day; T: time after treatment (minutes). ***P<.01 (*P*=.004, *P*=.001, *P*=.003, *P*=.004, and *P*=.007 at D1.T120, D1.T180, D2.T60, D2.T120, and D2.T180, respectively).

Similar to the pain intensity analysis, pain relief was evaluated both on days 1-2 and days 3-5. Differences in pain relief between baseline (prior to treatment initiation on day 1) and after study treatment use (60, 120, and 180 minutes) were assessed in the two groups. As shown in Figure 5, on the first 2 days, patients treated with Endocare experienced significantly higher pain relief than patients from the control group, except on day 1, 60 minutes (*t*_96.6312_=–1.149, *P*=.25).

Although not statistically significant, patients from the Endocare group had higher pain relief on day 3 and day 4 but not on day 5 when compared to the control group (Figure 5).

### Medications

During their participation, patients were allowed to use rescue pain medication and asked to report their use during the 5 days of treatment. Some patients stopped using the study treatments after day 2, but still reported their medication use until day 5. Considering days 1 and 2, only 1 patient in the control group stopped the study treatment but reported her drug intake on day 2.

Table 5 reports the overall medication intake (at least one single administration) for the major therapeutic classes, meaning that one patient may have taken multiple medications within the same therapeutic class. No difference was found between the two groups. Owing to this lack of difference, medications were not included in the mixed model for pain intensity assessment (ie, parsimony principle). Compared to that at baseline (Table 3), medication intake increased at day 1 and then decreased from day 2 onward, especially for anilides.

Table 6 illustrates the medication use (yes/no) on each day of participation. No difference was observed between the two treatment arms.

**Table 5:** Medication intake from day 1 to 5 of participation.

Medication		Day 1, n (%)			Day 2, n (%)			Day 3, n (%)			Day 4, n (%)			Day 5, n (%)	
		Control (n=51)	Endocare (n=51)	Control (n=50)		Endocare (n=49)	Control (n=32)		Endocare (n=37)	Control (n=23)		Endocare (n=29)	Control (n=22)		Endocare (n=20)
**Analgesics**															
	Anilides	15 (29.4)	12 (23.5)	13 (26.0)		9 (18.4)	9 (28.1)		10 (27.0)	6 (26.1)		4 (13.8)	5 (22.7)		3 (15.0)
	Natural opium alkaloids	0 (0.0)	2 (3.9)	1 (2.0)		2 (4.1)	0 (0.0)		2 (5.4)	0 (0.0)		1 (3.4)	0 (0.0)		1 (5.0)
	Opioids in combination with antispasmodics	0 (0.0)	2 (3.9)	0 (0.0)		1 (2.0)	0 (0.0)		1 (2.7)	0 (0.0)		1 (3.4)	0 (0.0)		1 (5.0)
	Opioids in combination with nonopioid analgesics	3 (5.9)	3 (5.9)	3 (6.0)		2 (4.1)	1 (3.1)		1 (2.7)	2 (8.7)		1 (3.4)	2 (9.1)		1 (5.0)
	Other analgesics and antipyretics	2 (3.9)	7 (13.7)	6 (12.0)		4 (8.2)	1 (3.1)		3 (8.1)	1 (4.3)		2 (6.9)	1 (4.5)		2 (10.0)
	Other opioids	1 (2.0)	3 (5.9)	2 (4.0)		2 (4.1)	2 (6.2)		2 (5.4)	1 (4.3)		2 (6.9)	1 (4.5)		2 (10.0)
	Salicylic acid and derivatives	0 (0.0)	0 (0.0)	0 (0.0)		0 (0.0)	0 (0.0)		0 (0.0)	0 (0.0)		0 (0.0)	0 (0.0)		0 (0.0)
Other antiemetics and antinauseants		0 (0.0)	0 (0.0)	0 (0.0)		0 (0.0)	0 (0.0)		0 (0.0)	0 (0.0)		0 (0.0)	0 (0.0)		0 (0.0)
Other antiepileptics		0 (0.0)	0 (0.0)	0 (0.0)		0 (0.0)	0 (0.0)		0 (0.0)	0 (0.0)		0 (0.0)	0 (0.0)		0 (0.0)
**Anti-inflammatory and antirheumatic products**															
	Fenamates	0 (0.0)	0 (0.0)	0 (0.0)		0 (0.0)	0 (0.0)		0 (0.0)	0 (0.0)		1 (3.4)	0 (0.0)		0 (0.0)
	Propionic acid derivates	10 (19.6)	13 (25.5)	9 (18.0)		12 (24.5)	8 (25.0)		8 (21.6)	3 (13.0)		9 (31.0)	5 (22.7)		3 (15.0)
Proton pump inhibitors (for acid-related disorders)		0 (0.0)	0 (0.0)	0 (0.0)		0 (0.0)	0 (0.0)		0 (0.0)	1 (4.3)		0 (0.0)	1 (4.5)		0 (0.0)
Other drugs for functional gastrointestinal disorders		4 (7.8)	4 (7.8)	4 (8.0)		2 (4.1)	3 (9.4)		1 (2.7)	1 (4.3)		1 (3.4)	3 (13.6)		1 (5.0)

**Table 6:** Principal medication use in patients adhering to study treatments.

Day		Analgesics, n (%)	NSAIDs^a^, n (%)	Gastrointestinal, n (%)	None, n (%)	*P* value	
**Day 1**							.95
	Control (n=51)	19 (37.3)	10 (19.6)	4 (7.8)	24 (47.1)		
	Endocare (n=51)	21 (41.2)	13 (25.5)	4 (7.8)	23 (45.1)		
**Day 2**							.66
	Control (n=50)	17 (34.0)	9 (18.0)	4 (8.0)	26 (52.0)		
	Endocare (n=49)	13 (26.5)	12 (24.5)	2 (4.1)	27 (55.1)		
**Day 3**							.74
	Control (n=29)	11 (37.9)	5 (17.2)	3 (10.3)	15 (51.8)		
	Endocare (n=34)	12 (35.3)	6 (17.6)	1 (2.9)	19 (55.9)		
**Day 4**							.24
	Control (n=21)	8 (38.1)	2 (9.5)	1 (4.8)	12 (57.1)		
	Endocare (n=24)	4 (16.7)	7 (29.2)	1 (4.2)	15 (62.5)		
**Day 5**							.78
	Control (n=18)	5 (27.8)	3 (16.7)	3 (16.7)	11 (61.1)		
	Endocare (n=18)	4 (22.2)	2 (11.1)	1 (5.6)	13 (72.2)		

^a^NSAID: nonsteroidal anti-inflammatory drug.

### Quality of Life

#### Patient Catastrophizing Scale

**Figure 6:**
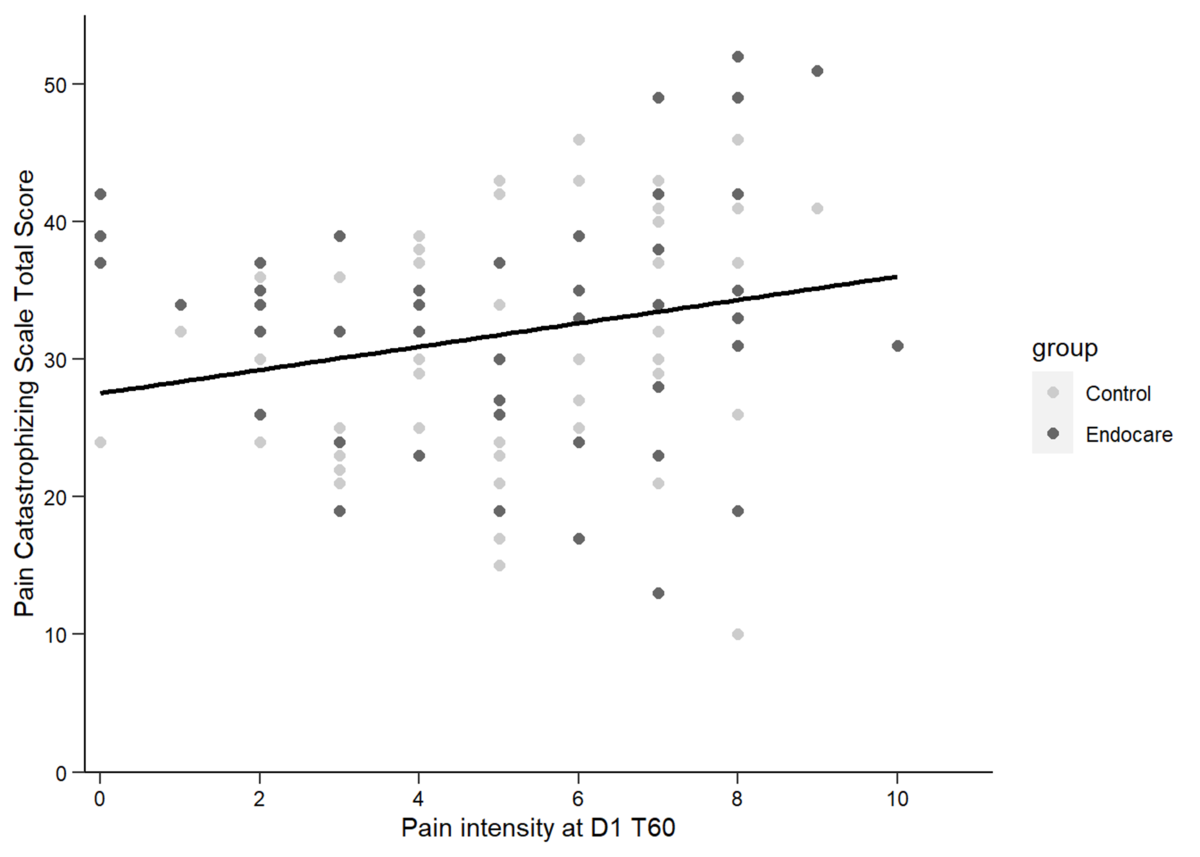
Correlation between total Pain Catastrophizing Score at baseline and pain intensity at day 1 60 minutes after treatment (D1 T60).

PCS was assessed to detect a potential bias in pain intensity/pain relief in enrolled patients. No difference of the PCS score between the two groups was observed at baseline (Endocare: mean 30.96, SD 9.00; control: mean 32.71, SD 8.73; *t*_99.9089_=–0.994, *P*=.32). However, a significant but minor correlation between the PCS score at baseline and pain intensity at day 1, 60 minutes was observed (*r*=0.22, *P*=.03; Figure 6), meaning that patients with a higher PCS score reported higher pain intensity at day 1, 60 minutes after treatment.

#### Fatigue and Stress

**Figure 7:**
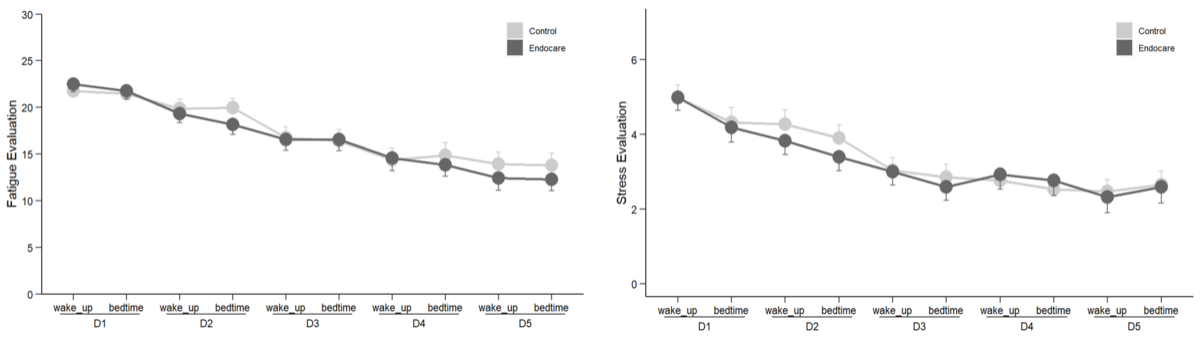
Fatigue and stress evaluation at wakeup and bedtime from day 1 to 5. D: day.

Both fatigue and stress measures were collected twice a day, at wakeup and bedtime. Fatigue was assessed using the Pichot scale, while changes in global stress were assessed using a visual analog scale. Both fatigue and stress were analyzed separately regarding the time of the day (wakeup or bedtime) using the same linear mixed model as used for pain intensity. Both patients in the Endocare and control groups declared reduced fatigue and reduced stress between day 1 and day 5 at wakeup and bedtime (Figure 7) with no difference between the groups.

Considering wakeup and bedtime, no effect of the group was observed for either the fatigue evaluation (wakeup: *F*_100_=0.180, *P*=.67; bedtime: *F*_99_=0.041, *P*=.84) or the stress evaluation (wakeup: *F*_100_=0.005, *P*=.94; bedtime: *F*_99_=0.138, *P*=.71), and there was also no significant group×day interaction for the fatigue evaluation (wakeup: *F*_382_=0.545, *P*=0.70; bedtime: *F*_379_=0.711, *P*=.59) or the stress evaluation (wakeup: *F*_384_=0.625, *P*=0.65; bedtime: *F*_377_=0.588, *P*=.67). However, significant effects were identified for day in the fatigue evaluation (wakeup: *F*_382_=19.860, *P*<.001; bedtime: *F*_379_=19.154, *P*<.001) and the stress evaluation (wakeup: *F*_384_=20.321, *P*<.001; bedtime: *F*_377_=12.197, *P*<.001).

#### Endometriosis Health Profile

The EHP-5 score is a quality-of-life score developed specifically for the population of women suffering from endometriosis. The score calculations are based on the answers to a standardized questionnaire, the interpretation of which results in the three variants of the EHP-5 score: total, core, and modular scores. No significant difference was found between the two groups for each of the three EHP-5 scores (Table 7).

**Table 7:** Endometriosis Health Profile (EHP-5) score evolution between inclusion and the end of study participation.

Group	EHP-5 total score, mean (SD)		EHP-5 core score, mean (SD)			EHP-5 modular score, mean (SD)	
	Day 0	Day 6	Day 0	Day 6	Day 0		Day 6
Endocare	44.8 (23.7)	44.0 (21.2)	61.1 (17.2)	57.8 (18.2)	31.2 (35.1)		32.4 (30.8)
Control	42.8 (21.8)	38.8 (21.8)	62.3 (17.2)	59.7 (15.4)	26.6 (33.0)		21.3 (34.5)

### PGIC and Satisfaction

Both PGIC and satisfaction were assessed at the end of the study (day 6), which were compared between the Endocare and control groups. Patients from the Endocare group reported higher improvement of their clinical condition compared to the control group (mean 5.12, SD 9.00 vs mean 4.24, SD 2.61; *t*_95.7429_=2.934, *P*=.004). In accordance with this result, patients from the Endocare group were significantly more satisfied than patients in the control group (mean 4.04, SD 1.32 vs mean 4.72, SD 1.29; *t*_97.949_=–2.597, *P*=.01).

### Adverse Events

No adverse events nor device deficiency were reported by the patients or detected by the investigating staff during the study period.

## Discussion

### Principal Results

#### Design

This study evaluated a new medical device software delivering a VR immersive experience specifically designed to alleviate and reduce pain in women suffering from chronic pelvic pain associated with endometriosis. Considering that chronic pain is a daily concern for affected patients [38], the study was designed to limit biases (randomized, double-blinded, placebo-controlled trial) and to assess the efficacy of the device when used at home by patients in total autonomy.

#### Pain Intensity and Pain Relief

Overall, pain intensity decreased from baseline (before study treatment initiation) until day 5 in the two study groups. Compared to the control group, significantly higher pain intensity reduction was observed in the Endocare group from the baseline up to day 3 (maximum on day 2 of 51.6% reduction in the Endocare group vs 21.2% in the control group). Concomitantly, a significant increase in pain relief was declared by the patients exposed to Endocare on days 1 and 2 compared to those using the digital sham control device. In contrast to usual pain medications, there are no particular safety risks associated with repeated use of a VR solution as confirmed in this trial.

These results confirmed the previously observed analgesic effects of the self-administered Endocare treatment (maximum pain intensity reduction of 42%) [24] and patients’ ability to use it at home on-demand in total autonomy. This study also confirmed the effect of VR itself (digital control) when associated with nature sounds, which is already used to reduce pain during surgery or for patients in intensive care [39,40]: its immersive effect has a direct impact on pain as observed in previous work [22,28,29,41-51]. Interestingly, compared to our previous study [24], the upgraded sham delivering the same audio/video content but in an immersive VR headset instead of a tablet achieved higher pain intensity reduction when used on a similar population.

#### Impact on Medication Use

During their participation, patients were free to use concomitant rescue pain medications as usual considering that the device efficacy may differ between participants. The objective was to evaluate patients’ adherence to a DTx, the integration of the device in their daily routine care, and its impact on pain medication intake. Unfortunately, only short-term data were collected and additional investigations would be needed to also demonstrate a long-term impact of the DTx on long-term medication use habits. Nevertheless, at equivalent medication use between groups, higher pain reduction was observed with Endocare.

Compared to baseline levels, patients declared increased use of medications. This was an expected result, as day 1 represents the patient’s first day of painful periods when they need pain relief the most. Despite the lack of difference in medication intake observed between the groups, looking at the analgesics used by the women to relieve their pain, the percentage of patients using any kind of analgesic medication decreased in the Endocare group by 14.7% (41.2% to 26.5%) against only 3.3% (37.3% to 34.0%) in the control group between day 1 and day 2 (Table 6). These results are consistent with the reductions in pain intensity observed in women between days 1 and 2 (up to 51% reduction with Endocare vs 23% in the control group). The decrease in pain intensity perceived by the women in the Endocare group was thus accompanied by a logical decrease in the use of analgesics, including opioids.

#### Stress/Fatigue and EHP-5

No difference in stress, fatigue, and quality of life was observed between the two study arms. Although evaluated in the context of the study, it was expected that the impact of the experimental treatments on quality of life (stress and fatigue) would be only minimally observable over a 5-day period, as this type of analysis is generally performed over longer periods (ie, weeks, months, years). As for medications, long-term data would be needed and more criteria should be investigated to assess the overall impact of the DTx on patients’ quality of life. Postmarket clinical studies should allow collecting such information to improve the solution.

### Comparative Assessment of Available Solutions

#### Home Versus Hospital Setting

Previous studies have shown that VR is effective in reducing acute pain in different types of populations or interventions [18-23]. While acute pain is treated in hospital settings, chronic pain is experienced on a daily scale and dedicated solutions that can be autonomously used at home without medical supervision are expected by patients [52]. To date, most studies have been conducted in a medical context under the supervision of the health care team [24-27] and only few have been conducted at patients’ homes with self-administered treatment [28].

Giving patients the opportunity to self-manage their pain treatment using an over-the-counter solution should increase their quality of life and have an impact on their pain medication intake, but should also have a beneficial impact on reducing medical staff and health care provider workload.

#### Pain Intensity Reduction

To our knowledge, only a few VR solutions to manage chronic pain exist that are also designed to be used at home. One device has been a game changer in the home-based treatment of chronic low back pain [28,50,51], which was the first SaMD cleared by the Food and Drug Administration to manage chronic low back pain using VR in 2021 [53]. Compared to a sham and considering predefined pain intensity reduction targets (30% and 50% pain intensity reduction), our device achieved very similar pain intensity reduction compared to that evaluated by Garcia and colleagues [28,53]. Interestingly, while the populations may be different, the technology used was comparable (Table 8).

**Table 8:** Comparison of pain intensity reduction between Endocare and the AppliedVR software as medical devices.

Device type		Pain intensity reduction	
		>30%	>50%
**Endocare days 1-2 (present study)**			
	Device	67.4%	47.8%
	Sham	31.9%	25.5%
**AppliedVR [53]**			
	Device	66.0%	46.0%
	Sham	41.0%	26.0%

#### Duration of the Effect

The duration of the analgesic effect of VR may vary between studies (up to several months posttreatment) [44], but the vast majority of VR solutions developed are focused on treating acute pain (eg, during surgery) in a limited time frame, in which the patients are exposed only once to the VR treatments.

The effect of Endocare evaluated in this study was assessed up to 3 and 4 hours in a previous study [24], confirming that pain reduction with a DTx can be comparable to some pharmacological analgesics and with only minor side effects (motion sickness). Interestingly, repeated administrations may have had a cumulative effect on pain reduction, similar to the effect of pharmacological therapies.

#### Accessibility for Patients

The medical device evaluated in this study relies both on a hardware and a proprietary software. It was designed to be accessible to all individuals and to treat their pelvic pain anywhere at any time. To complete the circle, the solution needs to be economically affordable. One must be aware that an important part of the costs is attributable to the hardware (VR headset manufacturer). As of today, the product is not yet on the market and multiple plans are envisaged, including (similar to pharmaceutical drugs) new “presentations/forms” to increase accessibility and usability, while minimizing the financial impact on patients.

### Limitations

The main limitation of this study is the drop of 18 patients after the randomization procedure for various reasons mentioned above. This event could have altered the comparability of the two groups, and it is the reason for which we compared the characteristics of patients included in the analysis. To check whether or not the comparability was altered, we also compared the 102 patients included in the analysis to the 18 patients who were not, and we observed their characteristics to be strictly comparable.

Moreover, from treatment initiation, while the patient cohort was quite stable on the first 2 days of participation, the participant number decreased continuously until day 5. In addition to the smaller group size, which affects the power of the statistical analysis, patients dropping out can affect patients’ randomization (from day 3 onward). The findings therefore describe the best available data. Reasons for stopping treatments were not collected and such a decrease did not allow a sufficient sample size to conclude on the main objective of the study from days 3 to 5.

Further, patients were exposed to only one 20-minute scenario: the same environment repeated up to twice a day may have induced some boredom in exposed patients and may have led some to discontinue the study treatments. This emphasizes the importance of patients’ engagement in this type of new home treatment and the need to develop strategies to ensure treatment adherence over time. Gamification and diversification may play a positive role in this aspect.

Finally, considering the objectives, this study was not conducted in a standardized hospital setting but rather at patients’ homes without strict medical supervision and based on declarative data. However, as a reminder, the device is intended to be used at home (over the counter) where and when patients need it the most. Chronic pain is constant and cannot only be treated during hospital consultations. Patients were also given the possibility to use the study treatments at any time of the day without a strict schedule, which may have had an impact on the treatment efficacy depending on their stress level and pain intensity that may vary during the day. The impact of the weekend versus weekdays has not been investigated and may have an influence on multiple parameters, including treatment adherence.

### Conclusions

This is the first study to report the home use, without medical supervision, of a VR solution designed to reduce pain in patients suffering from chronic pelvic pain associated with endometriosis. Although the study has some limitations due to its conduct at patients’ homes and not in a hospitalized, controlled environment, the results clearly demonstrated the feasibility and effectiveness of VR software use without medical supervision. The decrease of analgesic use and of pain intensity observed in the study provides evidence for the integration of VR in multimodal chronic pelvic pain treatment strategies and may help to avoid the potential opioid dependence observed in the long-term treatment of chronic pain. Long-term data would enable collecting more information about the effect of Endocare on analgesics use. Aside from its efficacy on reducing pain intensity, patients’ lifestyle will play an important role, as taking a pill remains easier than performing a 20-minute VR session.

Further studies are still needed to evaluate the efficacy of the VR software in other indications and to compare its efficacy versus traditional medications. Moreover, strategies should be implemented to increase adherence to the VR treatment as it may still be considered, despite its efficacy, as recreative and not therapeutic (eg, shorter exposition, various environments, new stimulation).

## Appendix

Multimedia Appendix 1 CONSORT-eHEALTH checklist (V 1.6.1).

## Abbreviations

DTx: digital therapeutic
EHP-5: Endometriosis Health Profile
PCS: Pain Catastrophizing Scale
PGIC: Patient's Global Impression of Change
SaMD: software as a medical device
VR: virtual reality

## Data Availability

For now, the data sets generated during and/or analyzed during this study are not publicly available as the product is not yet commercially available
